# Dynamics and Control of Diseases in Networks with Community Structure

**DOI:** 10.1371/journal.pcbi.1000736

**Published:** 2010-04-08

**Authors:** Marcel Salathé, James H. Jones

**Affiliations:** 1 Department of Biology, Stanford University, Stanford, California, United States of America; 2 Department of Anthropology, Stanford University, Stanford, California, United States of America; 3 Woods Institute for the Environment, Stanford University, Stanford, California, United States of America; Imperial College London, United Kingdom

## Abstract

The dynamics of infectious diseases spread via direct person-to-person transmission (such as influenza, smallpox, HIV/AIDS, etc.) depends on the underlying host contact network. Human contact networks exhibit strong community structure. Understanding how such community structure affects epidemics may provide insights for preventing the spread of disease between communities by changing the structure of the contact network through pharmaceutical or non-pharmaceutical interventions. We use empirical and simulated networks to investigate the spread of disease in networks with community structure. We find that community structure has a major impact on disease dynamics, and we show that in networks with strong community structure, immunization interventions targeted at individuals bridging communities are more effective than those simply targeting highly connected individuals. Because the structure of relevant contact networks is generally not known, and vaccine supply is often limited, there is great need for efficient vaccination algorithms that do not require full knowledge of the network. We developed an algorithm that acts only on locally available network information and is able to quickly identify targets for successful immunization intervention. The algorithm generally outperforms existing algorithms when vaccine supply is limited, particularly in networks with strong community structure. Understanding the spread of infectious diseases and designing optimal control strategies is a major goal of public health. Social networks show marked patterns of community structure, and our results, based on empirical and simulated data, demonstrate that community structure strongly affects disease dynamics. These results have implications for the design of control strategies.

## Author Summary

Understanding the spread of infectious diseases in populations is key to controlling them. Computational simulations of epidemics provide a valuable tool for the study of the dynamics of epidemics. In such simulations, populations are represented by networks, where hosts and their interactions among each other are represented by nodes and edges. In the past few years, it has become clear that many human social networks have a very remarkable property: they all exhibit strong community structure. A network with strong community structure consists of smaller sub-networks (the communities) that have many connections within them, but only few between them. Here we use both data from social networking websites and computer generated networks to study the effect of community structure on epidemic spread. We find that community structure not only affects the dynamics of epidemics in networks, but that it also has implications for how networks can be protected from large-scale epidemics.

## Introduction

Mitigating or preventing the spread of infectious diseases is the ultimate goal of infectious disease epidemiology, and understanding the dynamics of epidemics is an important tool to achieve this goal. A rich body of research [1],[2],[3] has provided major insights into the processes that drive epidemics, and has been instrumental in developing strategies for control and eradication. The structure of contact networks is crucial in explaining epidemiological patterns seen in the spread of directly transmissible diseases such as HIV/AIDS [1],[4],[5], SARS [6],[7], influenza [8],[9],[10],[11] etc. For example, the basic reproductive number R_0_, a quantity central to developing intervention measures or immunization programs, depends crucially on the variance of the distribution of contacts [1],[12],[13], known as the network degree distribution. Contact networks with fat-tailed degree distributions, for example, where a few individuals have an extraordinarily large number of contacts, result in a higher R_0_ than one would expect from contact networks with a uniform degree distribution, and the existence of highly connected individuals makes them an ideal target for control measures [7],[14].

While degree distributions have been studied extensively to understand their effect on epidemic dynamics, the community structure of networks has generally been ignored. Despite the demonstration that social networks show significant community structure [15],[16],[17],[18], and that social processes such as homophily and transitivity result in highly clustered and modular networks [19], the effect of such microstructures on epidemic dynamics has only recently started to be investigated. Most initial work has focused on the effect of small cycles, predominantly in the context of clustering coefficients (i.e. the fraction of closed triplets in a contact network) [20],[21],[22],[23],[24].

In this article, we aim to understand how community structure affects epidemic dynamics and control of infectious disease. Community structure exists when connections between members of a group of nodes are more dense than connections between members of different groups of nodes [15]. The terminology is relatively new in network analysis and recent algorithm development has greatly expanded our ability to detect sub-structuring in networks. While there has been a recent explosion in interest and methodological development, the concept is an old one in the study of social networks where it is typically referred to as a “cohesive subgroups,” groups of vertices in a graph that share connections with each other at a higher rate than with vertices outside the group [18]. Empirical data on social structure suggests that community structuring is extensive in epidemiological contacts [25],[26],[27] relevant for infectious diseases transmitted by the respiratory or close-contact route (e.g. influenza-like illnesses), and in social groups more generally [16],[17],[28],[29],[30]. Similarly, the results of epidemic models of directly transmitted infections such as influenza are most consistent with the existence of such structure [8],[9],[11],[31],[32],[33].

Using both simulated and empirical social networks, we show how community structure affects the spread of diseases in networks, and specifically that these effects cannot be accounted for by the degree distribution alone. The main goal of this study is to demonstrate how community structure affects epidemic dynamics, and what strategies are best applied to control epidemics in networks with community structure.

## Results

**Figure 1:**
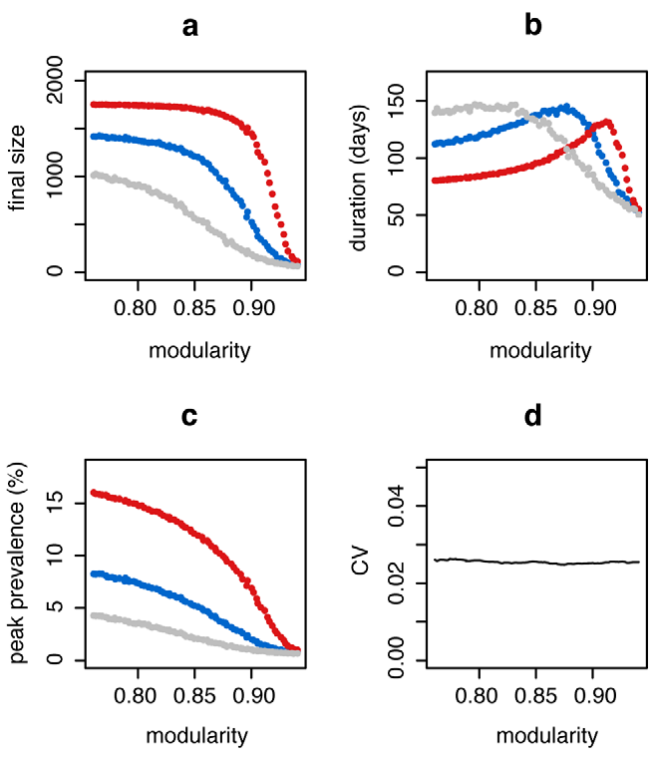
Effect of community structure, measured as modularity (*Q*) on epidemic dynamics. Panels show effect of community structure on (a) final size, (b) duration and (c) peak prevalence (i.e. maximum frequency of population infected). Each of the points represents the average of maximally 2000 simulation runs (only simulations with a final size of at least 2% of the population were included in calculating the averages). Error bars are omitted because the ranges are less than the size of the plotting points. The different colors represent different transmission rates: gray, *β* = 0.05 (R_0_≈*2.5*); blue, *β* = 0.06 (R_0_≈*3*); red, *β* = 0.08 (R_0_≈*4*). Panel (d) shows that the effect of a change in community structure on the squared coefficient of variation of the degree distribution (CV)^2^ is negligible.

We generate networks computationally with community structure by creating small subnetworks of locally dense communities, which are then randomly connected to one another. A particular feature of such networks is that the variance of their degree distribution is relatively low, and thus the spread of a disease is only marginally affected by it [34]. Running standard susceptible-infected-resistant (SIR) epidemic simulations (see Methods) on these networks, we find that the average epidemic size, epidemic duration and the peak prevalence of the epidemic are strongly affected by a change in community structure connectivity that is independent of the overall degree distribution of the full network (Figure 1). Note that the value range of *Q* shown in Figure 1 is in agreement with the value range of *Q* found in the empirical networks used further below, and that lower values of *Q* do not affect the results qualitatively (see Suppl. Mat. Figure S1).

**Figure 2:**
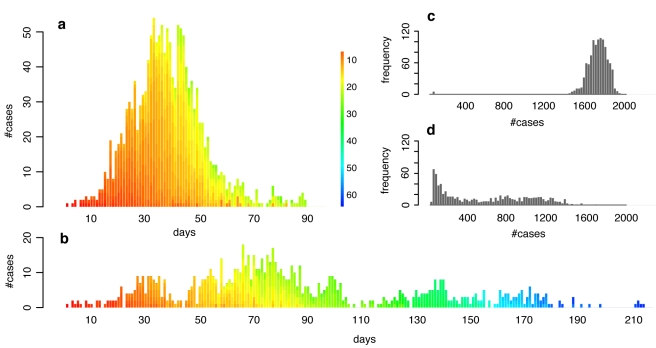
Typical incidence curves and distributions of final size in networks with medium and strong community structure. (a) and (b): Typical incidence curves of disease outbreaks in a network with medium community structure ((a): *Q≈0.76*) and a network with strong ((b): *Q≈0.9*) community structure (disease parameters equal to those in Figure 1 for the case where R_0_≈*3*). Each stacked bar represents the cumulative number of new cases during a given day. The color of a single infection case denotes the infection generation (initial case = 0), i.e. the number of hosts through which the infection has been passed on before infecting the current case. (c) and (d): Distribution of final size of simulations of disease outbreaks in a network with medium ((c), same contact network as in (a)) and strong ((d), same contact network as in (b)) community structure. Note that only simulations with a final size of at least 2% of the population were included in the distributions.

Epidemics in populations with community structure show a distinct dynamical pattern depending on the extent of community structure. In networks with strong community structure, an infected individual is more likely to infect members of the same community than members outside of the community. Thus, in a network with strong community structure, local outbreaks may die out before spreading to other communities, or they may spread through various communities in an almost serial fashion, and large epidemics in populations with strong community structure may therefore last for a long time. Correspondingly, the incidence rate can be very low, and the number of generations of infection transmission can be very high, compared to the explosive epidemics in populations with less community structure (Figures 2a and 2b). On average, epidemics in networks with strong community structure exhibit greater variance in final size (Figures 2c and 2d), a greater number of small, local outbreaks that do not develop into a full epidemic, and a higher variance in the duration of an epidemic.

**Figure 3:**
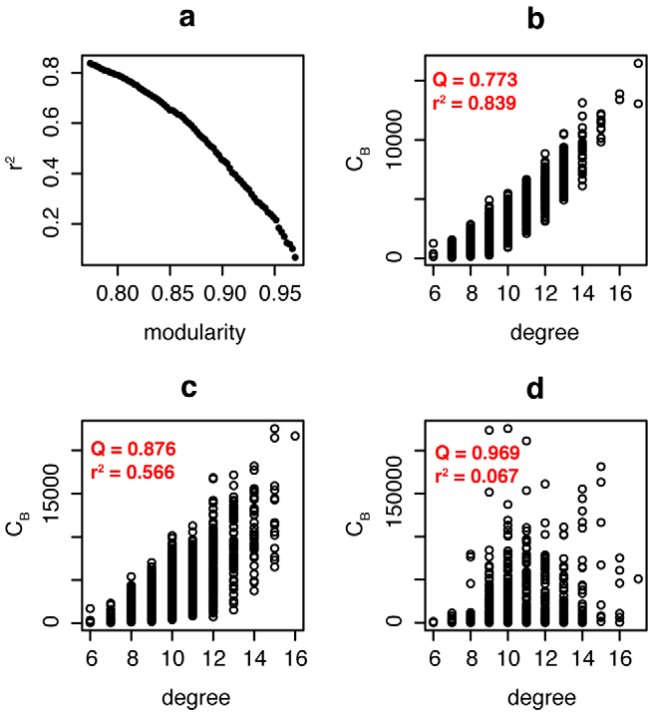
The breakdown of the correlation between degree and betweenness centrality (*C_B_*) with increasing community structure. (a) The correlation coefficient *r^2^* decreases rapidly as modularity increases. (b–d): Correlation between degree and betweenness in network with (b) medium, (c) strong and (d) very strong community structure.

**Figure 4:**
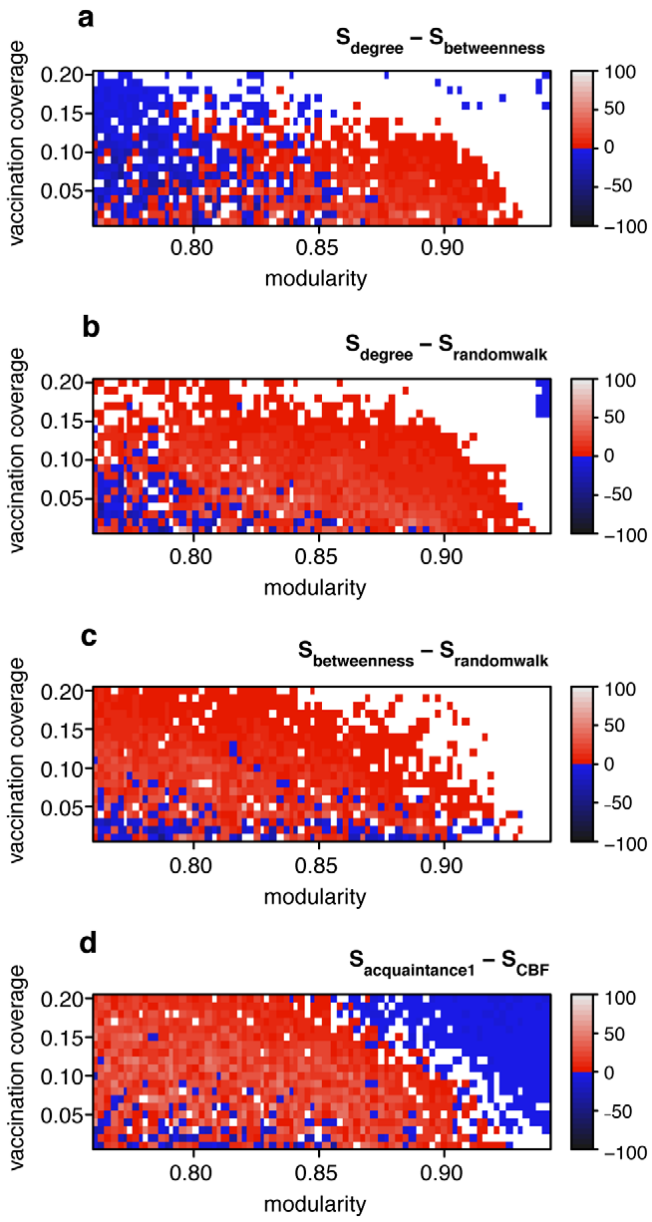
Assessing the efficacy of targeted immunization strategies based on deterministic and stochastic algorithms in the computationally generated networks. Color code denotes the difference in the average final size *S_m_* of disease outbreaks in networks that were immunized before the outbreak using method *m*. The top panel (a) shows the difference between the degree method and the betweenness centrality method, i.e. *S_degree_* − *S_betweenness_*. A positive difference (colored red to light gray) indicates that the betweenness centrality method resulted in smaller final sizes than the degree method. A negative difference (colored blue to black) indicates that the betweenness centrality method resulted in bigger final sizes than the degree method. If the difference is not bigger than 0.1% of the total population size, then no color is shown (white). Panel (a) shows that the betweenness centrality method is more effective than the degree based method in networks with strong community structure (*Q* is high). (b) and (c): like (a), but showing *S_degree_* − *S_randomwalk_* (in (b)) and *S_betweenness_* − *S_randomwalk_* (in (c)). Panels (b) and (c) show that the random walk method is the most effective method overall. Panel (d) shows that the community bridge finder (CBF) method generally outperforms the acquaintance method (with *n = 1*) except when community structure is very strong (see main text). Final epidemic sizes were obtained by running 2000 SIR simulations per network, vaccination coverage and immunization method.

In order to halt or mitigate an epidemic, targeted immunization interventions or social distancing interventions aim to change the structure of the network of susceptible individuals in such a way as to make it harder for a pathogen to spread [35]. In practice, the number of people to be removed from the susceptible class is often constrained for a number of reasons (e.g., due to limited vaccine supply or ethical concerns of social distancing measures). From a network perspective, targeted immunization methods translate into indentifying which nodes should be removed from a network, a problem that has caught considerable attention (see for example [36] and references therein). Targeting highly connected individuals for immunization has been shown to be an effective strategy for epidemic control [7],[14]. However, in networks with strong community structure, this strategy may not be the most effective: some individuals connect to multiple communities (so-called community bridges [37]) and may thus be more important in spreading the disease than individuals with fewer inter-community connections, but this importance is not necessarily reflected in the degree. Identification of community bridges can be achieved using the *betweenness centrality* measure [38], defined as the fraction of shortest paths a node falls on. While degree and betweenness centrality are often strongly positively correlated, the correlation between degree and betweenness centrality becomes weaker as community structure becomes stronger (Figure 3). Thus, in networks with community structure, focusing on the degree alone carries the risk of missing some of the community bridges that are not highly connected. Indeed, at a low vaccination coverage, an immunization strategy based on betweenness centrality results in fewer infected cases than an immunization strategy based on degree as the magnitude of community structure increases (Figure 4a). This observation is critical because the potential vaccination coverage for an emerging infection will typically be very low. A third measure, random walk centrality, identifies target nodes by a random walk, counting how often a node is traversed by a random walk between two other nodes [39]. The random walk centrality measure considers not only the shortest paths between pairs of nodes, but all paths between pairs of nodes, while still giving shorter paths more weight. While infections are most likely to spread along the shortest paths between any two nodes, the cumulative contribution of other paths can still be important [40]: immunization strategies based on random walk centrality result in the lowest number of infected cases at low vaccination coverage (Figure 4b and 4c).

**Figure 5:**
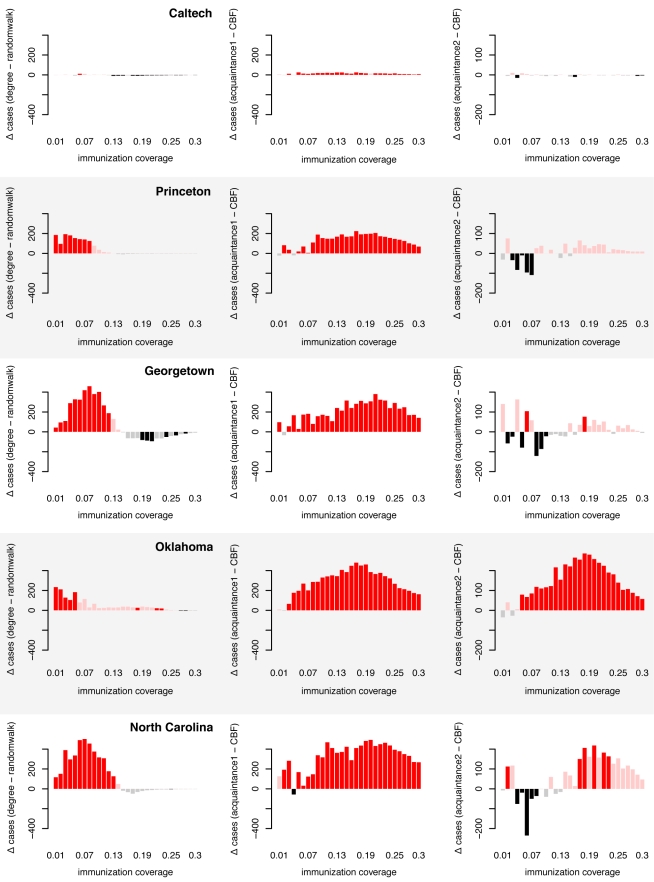
Assessing the efficacy of targeted immunization strategies in empirical networks based on deterministic and stochastic algorithms. The bars show the difference in the average final size *S_m_* of disease outbreaks (▵ cases) in networks that were immunized before the outbreak using method *m*. The left panels show the difference between the degree method and the random walk centrality method, i.e. *S_degree_* − *S_randomwalk_*. If the difference is positive (red bars), then the random walk centrality method resulted in smaller final sizes than the degree method. A negative value (black bars) means that the opposite is true. Shaded bars show non-significant differences (assessed at the 5% level using the Mann-Whitney test). The middle and right panels are generated using the same methodology, but measuring the difference between the acquaintance method (with *n = 1* in the middle column and *n = 2* in the right column, see Methods) and the community bridge finder (CBF) method, i.e. *S_acquaintance1_* − *S_CBF_* and *S_acquaintance2_* − *S_CBF_*. Again, positive red bars mean that the CBF method results in smaller final sizes, i.e. prevents more cases, than the acquaintance methods, whereas negative black bars mean the opposite. Final epidemic sizes were obtained by running 2000 SIR simulations per network, vaccination coverage and immunization method.

To test the efficiency of targeted immunization strategies on real networks, we used interaction data of individuals at five different universities in the US taken from a social network website [41], and obtained the contact network relevant for directly transmissible diseases (see Methods). We find again that the overall most successful targeted immunization strategy is the one that identifies the targets based on random walk centrality. Limited immunization based on random walk centrality significantly outperforms immunization based on degree especially when vaccination coverage is low (Figure 5a).

In practice, identifying immunization targets may be impossible using such algorithms, because the structure of the contact network relevant for the spread of a directly transmissible disease is generally not known. Thus, algorithms that are agnostic about the full network structure are necessary to identify target individuals. The only algorithm we are aware of that is completely agnostic about the network structure network structure identifies target nodes by picking a random contact of a randomly chosen individual [42]. Once such an acquaintance has been picked *n* times, it is immunized. The acquaintance method has been shown to be able to identify some of the highly connected individuals, and thus approximates an immunization strategy that targets highly connected individuals. We propose an alternative algorithm (the so-called *community bridge finder* (CBF) algorithm, described in detail in the Methods) that aims to identify community bridges connecting two groups of clustered nodes. Briefly, starting from a random node, the algorithm follows a random path on the contact network, until it arrives at a node that does not connect back to more than one of the previously visited nodes on the random walk. The basic goal of the CBF algorithm is to find nodes that connect to multiple communities - it does so based on the notion that the first node that does not connect back to previously visited nodes of the current random walk is likely to be part of a different community. On all empirical and computationally generated networks tested, this algorithm performed mostly better, often equally well, and rarely worse than the alternative algorithm.

It is important to note a crucial difference between algorithms such as CBF (henceforth called stochastic algorithms) and algorithms such as those that calculate, for example, the betweenness centrality of nodes (henceforth called deterministic algorithms). A deterministic algorithm always needs the complete information about each node (i.e. either the number or the identity of all connected nodes *for each node in the network*). A comparison between algorithms is therefore of limited use if they are not of the same type as they have to work with different inputs. Clearly, a deterministic algorithm with information on the full network structure as input should outperform a stochastic algorithm that is agnostic about the full network structure. Thus, we will restrict our comparison of CBF to the acquaintance method since this is the only stochastic algorithm we are aware of the takes as input the same limited amount of local information.

In the computationally generated networks, CBF outperformed the acquaintance method in large areas of the parameter space (Figure 4d). It may seem unintuitive at first that the acquaintance method outperforms CBF at very high values of modularity, but one should keep in mind that epidemic sizes are very small in those extremely modular networks (see Figure 1a) because local outbreaks only rarely jump the community borders. If outbreaks are mostly restricted to single communities, then CBF is not the optimal strategy because immunizing community bridges is useless; the acquaintance method may at least find some well connected nodes in each community and will thus perform slightly better in this extreme parameter space.

**Figure 6:**
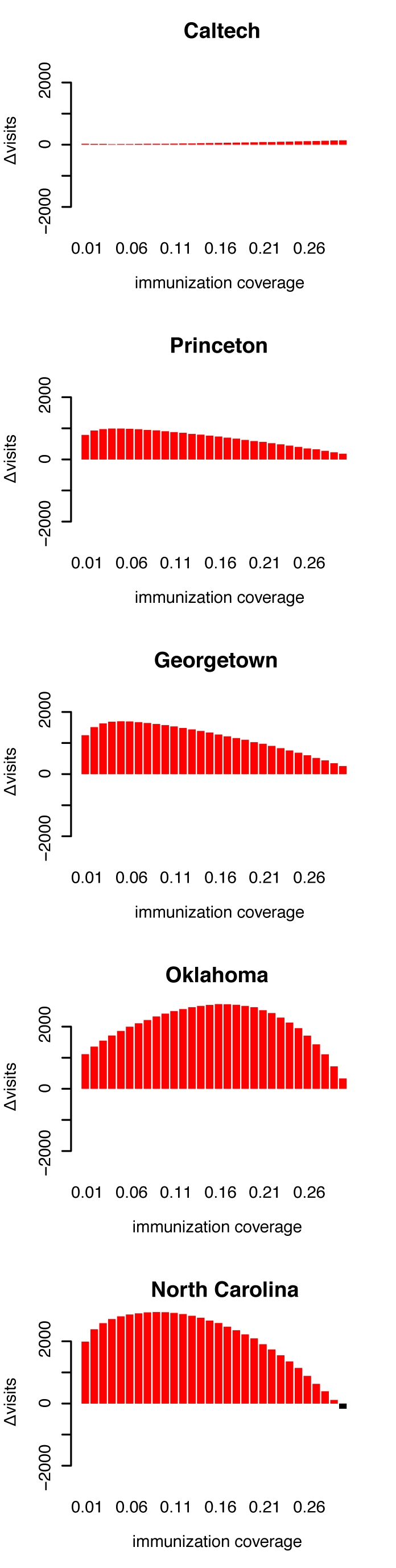
Assessing the speed of stochastic immunization algorithms acquaintance2 and CBF. The speed of an algorithm is assessed by counting the nodes that have to be visited by the algorithm until the desired vaccination coverage is achieved. Each visit is counted, even if the same node has been visited before. The bars show the difference of node visits (▵ visits) between the acquaintance2 method and the CBF method. Red bars mean the CBF method has visited fewer nodes - the difference is given by the height of the bar. A black bar indicates that the acquaintance2 methods has visited fewer nodes. With the exception of vaccination coverage 30% in the North Carolina network, the CBF method is always faster. (Data for speed comparison between acquaintance1 and CBF is not shown - the acquaintance1 method is always faster, but significantly less effective - see middle column in Figure 5).

In empirical networks, CBF did particularly well on the network with the strongest community structure (Oklahoma), especially in comparison to the similarly effective acquaintance method with *n = 2*. (Figure 5c). As immunization strategies should be deployed as fast as possible, the speed at which a certain fraction of the network can be immunized is an additional important aspect. We measured the speed of the algorithm as the number of nodes that the algorithm had to visit in order to achieve a certain vaccination coverage, and find that the CBF algorithm is faster than the similarly effective acquaintance method with *n = 2* at vaccination coverages <30% (see Figure 6).

## Discussion

A great number of infectious diseases of humans spread directly from one person to another person, and early work on the spread of such diseases has been based on the assumption that every infected individual is equally likely to transmit the disease to any susceptible individual in a population. One of the most important consequences of incorporating network structure into epidemic models was the demonstration that heterogeneity in the number of contacts (degree) can strongly affect how R_0_ is calculated [12],[13],[34]. Thus, the same disease can exhibit markedly different epidemic patterns simply due to differences in the degree distribution. Our results extend this finding and show that even in networks with the same degree distribution, fundamentally different epidemic dynamics are expected to be observed due to different levels of community structure. This finding is important for various reasons: first, community structure has been shown to be a crucial feature of social networks [15],[16],[17],[19], and its effect on disease spread is thus relevant to infectious disease dynamics. Furthermore, it corroborates earlier suggestions that community structure affects the spread of disease, and is the first to clearly isolate this effect from effects due to variance in the degree distribution [43]. Second, and consequently, data on the degree distribution of contact networks will not be sufficient to predict epidemic dynamics. Third, the design of control strategies benefits from taking community structure into account.

An important caveat to mention is that community structure in the sense used throughout this paper (i.e. measured as modularity *Q*) does not take into account explicitly the extent to which communities overlap. Such overlap is likely to play an important role in infectious disease dynamics, because people are members of multiple, potentially overlapping communities (households, schools, workplaces etc.). A strong overlap would likely be reflected in lower overall values for *Q*; however, the exact effect of community overlap on infectious disease dynamics remains to be investigated.

Identifying important nodes to affect diffusion on networks is a key question in network theory that pertains to a wide range of fields and is not limited to infectious disease dynamics only. There are however two major issues associated with this problem: (i) the structure of networks is often not known, and (ii) many networks are too large to compute, for example, centrality measures efficiently. Stochastic algorithms like the proposed CBF algorithm or the acquaintance method address both problems at once. To what extent targeted immunization strategies can be implemented in a infectious diseases/public health setting based on practical and ethical considerations remains an open question. This is true not only for the strategy based on the CBF algorithm, but for most strategies that are based on network properties. As mentioned above, the contact networks relevant for the spread of infectious diseases are generally not known. Stochastic algorithms such as the CBF or the acquaintance method are at least in principle applicable when data on network structure is lacking.

Community structure in host networks is not limited to human networks: Animal populations are often divided into subpopulations, connected by limited migration only [44],[45]. Targeted immunization of individuals connecting subpopulations has been shown to be an effective low-coverage immunization strategy for the conservation of endangered species [46]. Under the assumption of homogenous mixing, the elimination of a disease requires an immunization coverage of at least 1-1/R_0_ [1] but such coverage is often difficult or even impossible to achieve due to limited vaccine supply, logistical challenges or ethical concerns. In the case of wildlife animals, high vaccination coverage is also problematic as vaccination interventions can be associated with substantial risks. Little is known about the contact network structure in humans, let alone in wildlife, and progress should therefore be made on the development of immunization strategies that can deal with the absence of such data. Stochastic algorithms such as the acquaintance method and the CBF method are first important steps in addressing the problem, but the large difference in efficacy between stochastic and deterministic algorithms demonstrates that there is still a long way to go.

## Methods

### SIR simulations

To investigate the spread of an infectious disease on a contact network, we use the following methodology: Individuals in a population are represented as nodes in a network, and the edges between the nodes represent the contacts along which an infection can spread. Contact networks are abstracted by undirected, unweighted graphs (i.e. all contacts are reciprocal, and all contacts transmit an infection with the same probability). Edges always link between two distinct nodes (i.e. no self loops), and there must be maximally one edge between any single pair of nodes (i.e no parallel edges). Each node can be in one of three possible states: (S)usceptible, (I)nfected, or (R)esistant/immune (as in standard SIR models). Initially, all nodes are susceptible.

Simulations with immunization strategies implement those strategies before the first infection occurs. Targeted nodes are chosen according to a given immunization algorithm (see below) until a desired immunization coverage of the population is achieved, and then their state is set to resistant.

After this initial set-up, a random susceptible node is chosen as patient zero, and its state is set to infected. Then, during a number of time steps, the initial infection can spread through the network, and the simulation is halted once there are no further infected nodes. At each time step (the unit of time we use is one day, *i.e.* a time step is one day), an infected node can get infected with probability *1−exp(−βi)*, where *β* is the transmission rate from an infected to a susceptible node, and *i* is the number of infected neighboring nodes. At each time step, infected nodes recover at rate *γ*, i.e. the probability of recovery of an infected node per time step is *γ* (unless noted otherwise, we use *γ = 0.2*). If recovery occurs, the state of the recovered node is toggled from infected to resistant. Unless mentioned otherwise, the transmission rate *β* is chosen such that *R_0_*∼(*β*/γ) * *d≈3* where *d* is the mean network degree, i.e the average number of contacts per node. For the networks used here, this approximation is in line with the result from static network theory [47], *R_0_∼T(<k^2^>/<k>−1)*, where *<k>* and *<k^2^>* are the mean degree and mean square degree, respectively, and where *T* is the average probability of disease transmission from a node to a neighboring node, i.e. *T≈β/γ*. Note that the variation in the degree is too small to be of relevance here (see further below and Figure 1d). The reason we chose *γ = 0.2* (i.e. an average length of infectious period of 5 days) and *R_0_≈3* in most simulations (unless mentioned otherwise) is that these parameter values reflect, very roughly, some of the most widespread infectious diseases to which our study is relevant (i.e. flu-like infectious diseases that are transmitted directly from person to person by the respiratory or close-contact route [8],[9],[48],[49],[50]).

After a simulation, we record the total number of cases infected (the epidemic size), the maximum frequency of infection at any point during the simulation (the peak prevalence), and the number of days that have passed between the first infected case and the simulation stop (the duration of the epidemic).

### Generation of network with community structure

In order to understand the effect of community structure, we generated networks with 2000 nodes from scratch with varying degrees of community structure. The strength of community structure is generally measured as network modularity *Q*, which is defined as
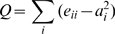
where *e_ij_* is the fraction of all edges in the network that link nodes in community *i* to nodes in community *j*, and
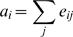

[15]. Thus, *a_i_* represents the fraction of edges in the network that connect to nodes in community *i*. If edges were to fall between nodes without any regard for communities, we would have *e_ij_ = a_i_ a_j_*, and thus *Q = 0*. There are numerous methods to calculate the value of *Q* for a given network, and the development of more accurate and efficient methods is still a very active research field. In particular, one has to be careful when comparing values of *Q* because some measures are normalized while others or not [51]. We have used the spin glass method introduced by Reichhardt and Bornholdt [52] to measure *Q* throughout this manuscript.

To generate networks with community structure, we initialize a network by creating 50 small-world communities (as found in various social networks, see e.g. ref. [53]) of 40 nodes using the Watts-Strogatz algorithm [54] such that each node has exactly 8 edges connecting to nodes of the same community. We then add 2000 edges randomly between randomly chosen nodes, making sure that the edges fall *between* communities only. Thus, we create a graph with 2000 nodes and 10000 (*i.e.* (2000+50 * 40 * (8/2))) undirected edges where one out of five edges falls between communities. The average degree of the network is 10, which is in line with recent reports on social contact patterns [55]. Starting from this initial network where *Q≈0.76*, we create networks with increasing community structure by rewiring *between-community* edges so that they become *within-community* edges. More precisely, at each rewiring step, we (i) randomly choose a between-community edge, (ii) randomly choose one of the two communities that the edge connects, (iii) pick a random node of the chosen community, and (iv) rewire the edge by detaching it from the node of the community that was not chosen in step (ii), and attaching it to the new node in the community that was chosen in step (iii). At all times, edges must always fall between two distinct nodes, and there can only be one edge between any two pair of nodes. We've also tested if all networks thus created consist of only a single connected component (they do).

The quantity *(CV)^2^* is the square of the coefficient of variation in degree (i.e. the squre of the ratio of the standard deviation of degree to the mean degree, where degree is defined as the number of edges incident to a node). *(CV)^2^* is important for the spread of infectious diseases since it is known that
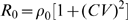
where *ρ_0_* is the value of *R_0_* under the assumption of a homogenous network (i.e. no variance in the degree distribution) [1],[56].

### Empirical networks

We used the network data collected on the social network website Facebook (www.facebook.com) by Traud et al. [41]. The data contains the friendship network at five US universities, where nodes represent individuals (i.e. members of the university), and edges represent friendship links between two individuals. Additionally, the data includes covariate information (if available) about the individuals, such as the gender of the individual, the dormitory residence, major (field of specialization) etc. While such friendship network data are interesting for various reasons, they do not necessarily reflect the contact network relevant for the spread of infectious diseases. Clearly, a friendship connection between two individuals on a social network website does not necessarily mean that there is also a connection between the two individuals in the contact network relevant to the spread of infectious diseases.

Thus, in order to obtain contact network data that are relevant for the spread of infectious diseases transmitted directly from person to person by the respiratory or close-contact route, we make the following assumptions: Individuals who have a friendship relation in the network, and who either (a) have the same dormitory residence, or (b) who major in the same field and the same class year, are likely to be in close enough physical contact on a regular basis as to be able to transmit an infection to each other. Thus, using the raw friendship data and the available information on dormitory residence, major, and class year, we extract the subgraph which reflects our assumptions. Having extracted the subgraph, we remove all nodes who are not part of the largest connected component (i.e. small subgraphs that are not part of the larger network). The networks thus reduce to the following contact networks:

- Caltech (620 nodes and 7,255 edges, *Q = 0.788*)
- Princeton (5,112 nodes and 28,684 edges, *Q = 0.753*)
- Georgetown (7,651 nodes and 79,799 edges, *Q = 0.662*)
- Oklahoma (10,386 nodes and 163,225 edges, *Q = 0.914*)
- North Carolina (13,081 nodes, 88,266 edges, *Q = 0.812*)

We note that the modularity *Q* of these networks is within the range of modularities measured in the computationally generated networks (see for example Figure 1), with the exception of one network (Georgetown). Clearly, these networks will contain contacts that are not relevant for the spread of diseases (false positives) - at the same time, they will also miss some relevant contacts (false negatives). However, given the accuracy and amount of data, these networks are well suited to study the spread of infectious diseases on human contact networks, in particular for diseases transmitted directly from person to person by the respiratory or close-contact route. Degree distributions of these networks are shown in Suppl. Mat. Figure S2.

### Immunization algorithms

The algorithms used to identify nodes can be divided into two classes: deterministic and stochastic algorithms. Deterministic algorithms require the complete information about each node (i.e. either the number or the identity of all connected nodes *for each node in the network*), and they rank nodes by processing that information by a procedure specific to that algorithm. Target nodes are then chosen by their rank (from high to low). Thus, for a given network structure, deterministic algorithms always give the same result, i.e. they identify the same target nodes (except for random choices when two nodes have exactly the same rank). Stochastic algorithms, on the other hand, do not require such detailed structural information - they identify target nodes by collecting information locally from *randomly* chosen nodes in the network. These algorithms represent the type of investigation-related information in actual epidemics. We will now describe a number of deterministic and stochastic algorithms as we have used them in the main text.

#### Deterministic algorithms

We identifiy target nodes by ranking nodes to one of the three following criteria: degree, betweenness centrality, and random-walk centrality.

The degree of a node simply denotes the number of edges incident to a node.

The betweenness centrality *C_B_(i)* of a node *i* is defined as
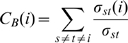
where *s*, *t* and *i* are distinct nodes of the graph, *σ_st_* is the total number of shortest paths between nodes *s* and *t*, and *σ_st_(i)* is the number of those shortest paths that go through node *i* [38].

The random-walk centrality of a node *i* is a measure based on random walks, counting how often the node *i* is traversed by a random walk between any pair of nodes *s* and *t*. Following Newman [39], we rank nodes according to the random-walk measure
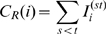
where
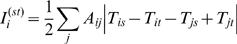
for *i*≠*s*, *t*. Here, *A_ij_* is the element in the adjacency matrix of the graph (0 or 1 in our case), and *T_is_* is the element in the voltage matrix which is calculated as described in detail in Newman [39].

Nodes are ranked according to the measure chosen (i.e degree, betweenness centrality, or random-walk centrality). We then immunize nodes going from high to low rankings, until the desired immunization coverage is achieved.

#### Stochastic algorithms

We use two stochastic algorithms to identify target nodes without knowledge of the full network structure. In the algorithms described below, targets are identified and immunized if they have not been immunized before.

The first algorithm, acquaintance immunization, has been described by Cohen et al. [42] and it works as follows: pick a random node *v_0_*, and then pick a random acquaintance *v_1_*, i.e. a randomly picked neighboring node of *v_0_*. Immunize nodes that have been referred to as acquaintances at least *n* times until the desired immunization coverage is achieved. In the case *n* = 1, every acquaintance will be immunized immediately. The acquaintance strategy has been shown to identify highly connected individuals, particularly in fat-tailed networks (such as so-called scale-free networks).

**Figure 7:**
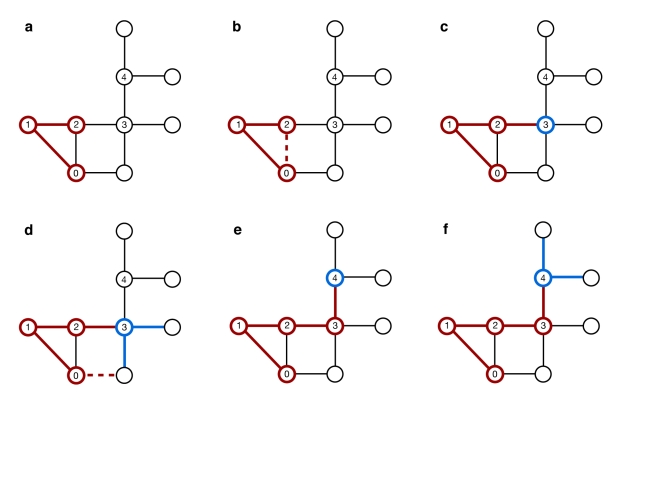
Sketch of the community bridge finder algorithm. (a) A random walk follows the path starting from v_0_ to v_1_ and v_2_, at which point it starts checking for connections of v_2_ to v_0_ and v_1_. (b) Since there are more than one connections (v_2_-v_1_ and v_2_-v_0_), the walk continues to v_3_. (c) Except the obvious v_3_-v_2_, there are no connections from v_3_ to any of the previously visited nodes, so v_2_ is a potential target. (d) The algorithm then picks two random neighbors of v_3_ to check for connections to previously visited nodes - and finds one (to v_0_). (e) Hence, v_2_ is dismissed as a potential target, and the random walk continues to v_4_. Again, v_4_ does not back-connect to any previously visited node (except, of course, to v_3_), and thus v_3_ is identified as a potential target - (f) thus again, two random neighboring nodes are picked to check for connections to previously visited nodes. Since no back connections can be found, v_3_ is identified as a target and immunized.

We propose another strategy, the community-bridge-finder (CBF) strategy, which rests on the observation that some individuals act as bridges between communities. The goal of the CBF algorithm is to identify such individuals based on random walks, without knowledge of the network structure, and thus without knowledge of the communities in a network. The algorithm works as follows: pick a random node *v_i = 0_* and follow a random path (one node at a time, with the only condition that a node has not been visited by the random walk before). At every node *v_i≥2_*, check if there is more than one connection from *v_i_* to any of the visited nodes (the requirement for more than one connection stems from the simple fact that every node *v_i_* will have at least one connection to *v_i−1_*). If there is just one back connection (i.e. from *v_i_* to *v_i−1_*), a potential target *v_i−1_* has been identified. As an additional check, pick two random neighboring nodes of *v_i_* (other than *v_i−1_*) and check for connections back to the previously visited nodes *v_j<i_*. If such connections exist, *v_i−1_* is not a potential target - continue the random walk at *v_i−1_*. If no such connections exist, immunize the potential target. Discard all information about visits, and start again at a randomly picked node *v_0_*. A schematic sketch of the algorithm is outlined in Figure 7.

An algorithmic search for community bridges as described above can potentially take a very long time, depending on the structural features of the network. For example, the frequency of nodes that can potentially meet the immunization requirement set by the algorithm might be smaller than the desired immunization coverage. To prevent endless searches for community bridges, two additional checks are implemented. First, the number of nodes in any running random path does not exceed 10 (this is implemented using a first-in-first-out list that keeps track of the visited nodes). Second, we keep track of all nodes visited, and if a node has been visited at least *k* times (on any random walk), it will be immunized. In all results presented in this manuscript, we use *k = 2*.

## Supporting Information

Figure S1 Results from simulations with the same parameters and settings as Figure 1a in the main text, but based on networks with lower community structure. The initial creation of these networks was identical to those created for Figure 1 in the main text (see description in Methods in the main text), but rather than rewiring between-community edges and turn them into within-community edges, we randomly rewired within-community edges in the following way: at each rewiring step, we (i) randomly choose a within-community edge, (ii) randomly choose one of the two nodes, (iii) pick a random node in the network, and rewire the edge by detaching it from the node that was not chosen in step (ii), and attaching it to the new node that was chosen in step (iii). At all times, edges must always fall between two distinct nodes, and there can only be one edge between any two pair of nodes. Note that this algorithm is essentially the reverse of the algorithm used to create networks with increased community structure in the main text.
(2.38 MB TIF)

Figure S2 Degree distributions of the empirical networks used in the main text. Main panels show cumulative frequency distributions; insets show non-cumulative frequency distributions.
(2.24 MB TIF)
