# Supplementary figures and images for: Dynamics and Control of Diseases in Networks with Community Structure
Source: PLoS Comput Biol. 2010 Apr 8;6(4):e1000736. doi: 10.1371/journal.pcbi.1000736 (PMC2851561; doi:10.1371/journal.pcbi.1000736)

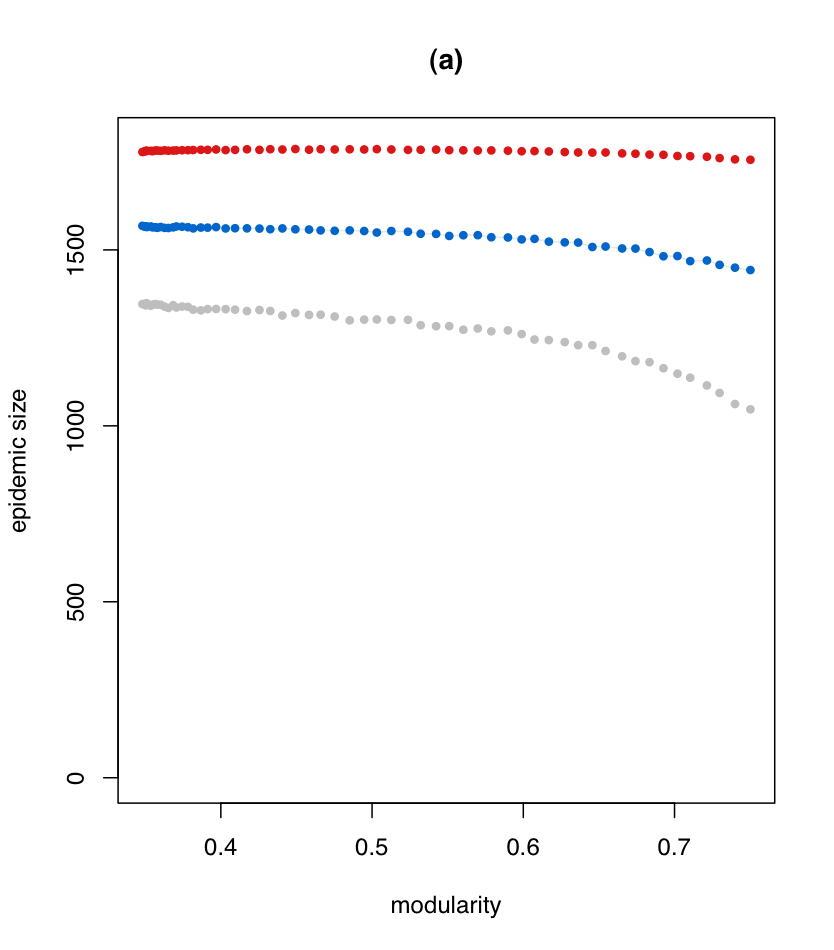

Supplement: Figure S1 — Results from simulations with the same parameters and settings as Figure 1a in the main text, but based on networks with lower community structure. The initial creation of these networks was identical to those created for Figure 1 in the main text (see description in Methods in the main text), but rather than rewiring between-community edges and turn them into within-community edges, we randomly rewired within-community edges in the following way: at each rewiring step, we (i) randomly choose a within-community edge, (ii) randomly choose one of the two nodes, (iii) pick a random node in the network, and rewire the edge by detaching it from the node that was not chosen in step (ii), and attaching it to the new node that was chosen in step (iii). At all times, edges must always fall between two distinct nodes, and there can only be one edge between any two pair of nodes. Note that this algorithm is essentially the reverse of the algorithm used to create networks with increased community structure in the main text. (2.38 MB TIF) [file pcbi.1000736.s001.tif]

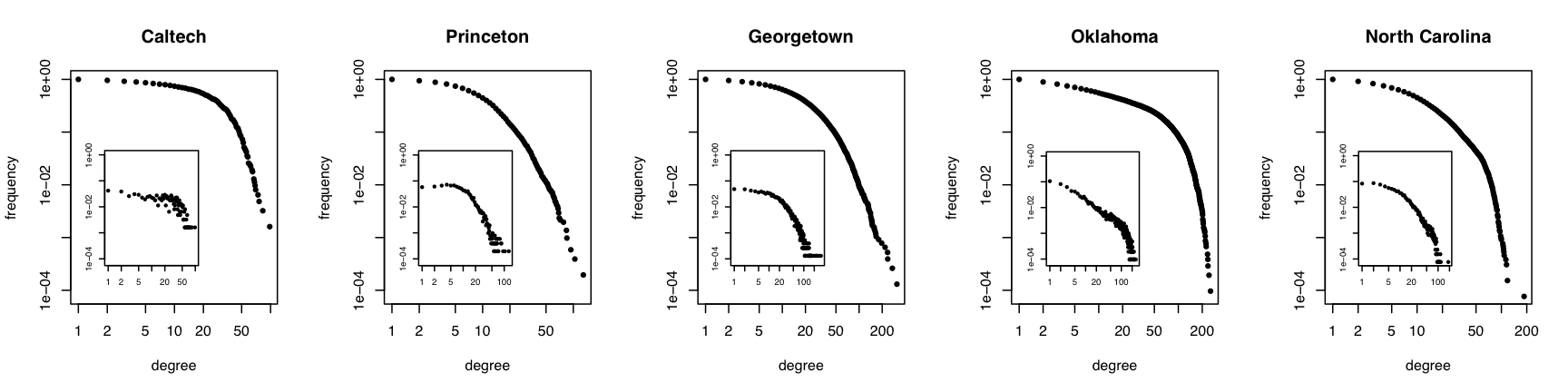

Supplement: Figure S2 — Degree distributions of the empirical networks used in the main text. Main panels show cumulative frequency distributions; insets show non-cumulative frequency distributions. (2.24 MB TIF) [file pcbi.1000736.s002.tif]
